# Completion lobectomy after anatomical segmentectomy

**DOI:** 10.1093/icvts/ivab323

**Published:** 2021-12-28

**Authors:** Satoshi Takamori, Hiroyuki Oizumi, Jun Suzuki, Katsuyuki Suzuki, Hikaru Watanabe, Kaito Sato

**Affiliations:** Department of Surgery II, Faculty of Medicine, Yamagata University, Yamagata, Japan

**Keywords:** Completion lobectomy, Video-assisted thoracoscopic surgery, Segmentectomy

## Abstract

**OBJECTIVES:**

Completion lobectomy (CL) after anatomical segmentectomy in the same lobe can be complicated by severe adhesions around the hilar structures and may lead to fatal bleeding and lung injury. Therefore, we aimed to investigate the perioperative outcomes of CL after anatomical segmentectomy.

**METHODS:**

Among 461 patients who underwent anatomical segmentectomy (thoracotomy, 62 patients; thoracoscopic surgery, 399 patients) between January 2005 and December 2019, data of patients who underwent CL after segmentectomy were extracted and analysed in this study.

**RESULTS:**

Eight patients underwent CL after segmentectomy. CL was performed via video-assisted thoracic surgery in 3 patients and thoracotomy in 5 patients. In each case, there were moderate to severe adhesions. Four patients required simultaneous resection of the pulmonary parenchyma and pulmonary artery. Thoracotomy was not required after thoracoscopic surgery in any patient. Two patients experienced complications (air leakage and arrhythmia). The median duration of hospitalization after CL was 6 (range, 5–7) days. No postoperative mortality or recurrence of lung cancer was observed. All the patients with lung cancer were alive and recurrence-free at the time of publication.

**CONCLUSIONS:**

Although individual adhesions render surgery difficult, CL after anatomical segmentectomy shows acceptable perioperative outcomes. However, CL by video-assisted thoracoscopic surgery may be considered on a case-by-case basis depending on the initial surgery.

**Figure:**
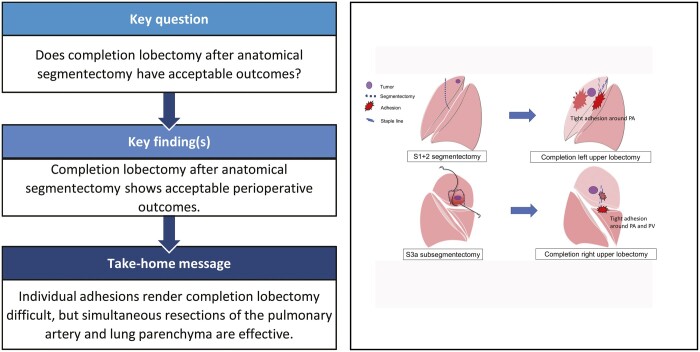
Patient selection flowchart.

## INTRODUCTION 

The number of indications for anatomical segmentectomy has recently increased because of its effectiveness and good postoperative lung function. The results of sublobar resection have been reported as being relatively good, and segmentectomy is a potentially acceptable technique for small-sized lung cancers and metastatic tumours [1–8]. Outcomes after intentional segmentectomy are favourable in comparison with those after standard lobectomy in patients with Stage I non-small-cell lung cancer (NSCLC). In addition, recent large-scale retrospective studies [9, 10] and meta-analyses [11] have reported no statistically significant differences in disease-specific or disease-free survival, overall survival (OS) or recurrence rates between the 2 techniques. An improvement in the survival of patients with lung cancer who undergo surgery may lead to a potential risk of developing a second or even a third NSCLC [12]. Therefore, completion lobectomy (CL), comprising resection of the same pulmonary lobe after sublobar resection, is considered in cases involving a second primary lung cancer and/or metastatic lung cancer after segmentectomy. CL after diagnostic wedge resection using video-assisted thoracoscopic surgery (VATS) may be feasible and safely performed [13, 14]. Although cases of CL after anatomical segmentectomy have been reported [15–17], performing CL after segmentectomy in the same lobe can be complicated by severe adhesions around the hilar structures, especially around the pulmonary artery (PA) and lung parenchyma [16, 17]. Fatal bleeding and lung injury may also occur.

Therefore, we aimed to investigate the perioperative outcomes of CL after anatomical segmentectomy.

## PATIENTS AND METHODS

### Patient selection

The retrospective study protocol was approved by the Ethics Committee of the Faculty of Medicine, Yamagata University (#2020-S-42, 31 August 2020), and the requirement to obtain written informed consent from each patient was waived by the hospital’s Institutional Review Board.

**Figure 1:**
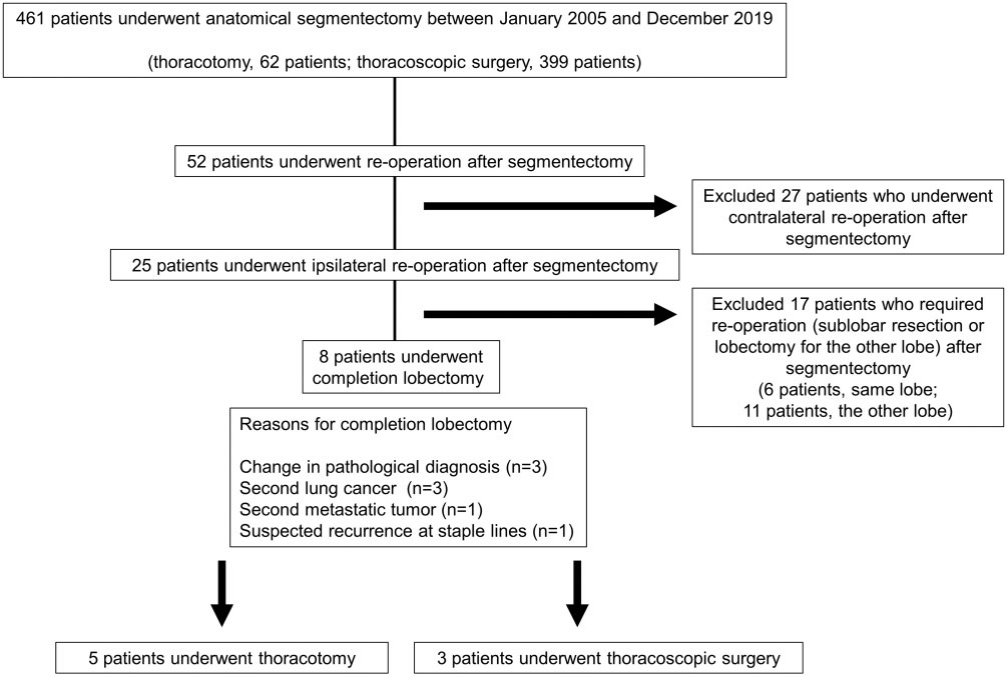
Patient selection flowchart.

We retrospectively reviewed the records of 461 patients (thoracotomy, 62 patients; thoracoscopic surgery, 399 patients) who underwent anatomical segmentectomy at our institute between January 2005 and December 2019 (Fig. 1). The indication for CL was adequate cardiopulmonary function for lobectomy in patients who required the surgery to manage new lesions (lung cancer, metastatic tumour or local recurrence) in the same lobe after segmentectomy. Preoperative three-dimensional simulation was used to confirm the tumour and hilum structure. Three-dimensional computed tomography (CT) was performed by the surgeon in all cases. Indications for anatomical segmentectomy at our hospital were: NSCLC ≤2 cm with a consolidation/tumour ratio ≤20%, solid NSCLC ≤1 cm and indeterminate nodules ≤1.5 cm. The selected patients had good pulmonary function and could tolerate a lobectomy to manage their disease. On the other hand, patients who could not tolerate lobectomy opted for segmentectomy to manage their disease. Patients with deep metastatic and benign lung tumours, in whom partial resection was not possible, underwent segmentectomy with adequate margins. Data on the clinical characteristics and medical history of the patients were obtained.

In cases of suspected malignancy, resection lines were planned such that the surgical margin was larger than the tumour diameter (≥1 cm, even for tumours smaller than 1 cm). In single-segment resection cases, wherein preserving the margin was difficult, we performed extended segmentectomy by dissecting the parenchyma across the intersegmental vein or combined segmentectomy using the adjacent subsegments. We performed lymph node sampling instead of dissection in cases of lung cancer presenting with pure ground-glass nodules and mediastinal lymph node dissection in cases of solid tumours and tumours with partially solid ground-glass nodules. In addition, in cases of small metastatic tumours, we performed lymph node sampling. However, if hilar lymph node metastasis was suspected, lobectomy was performed.

We evaluated the indications for segmentectomy and CL, interval between segmentectomy and CL, surgical outcomes of the CL, approach, operative procedure, extent and degree of adhesions, PA injury, use of fibrin glue, postoperative drainage duration, postoperative hospitalization duration, postoperative complications, mortality, OS and lung cancer-specific survival (LCSS).

### Follow-up

Follow-up visits were scheduled every 3 or 6 months until 12 months postoperatively and every 6 months or yearly thereafter. Standard follow-up evaluation comprised history evaluation, clinical examination and chest radiography. A routine CT scan of the chest was obtained every 6 months or yearly. The median follow-up time was 5.5 years for the entire cohort.

### Recurrence of malignant tumours

Locoregional recurrence was defined as evidence of a tumour (with the same histologic type as the primary tumour) within the same lobe or ipsilateral hilar and mediastinal lymph nodes. Distant recurrence was defined as evidence of a tumour (with the same histologic type as the primary tumour) in another lobe, in the pleural space or elsewhere outside the hemithorax.

### Statistical analyses

Continuous data are presented as median with interquartile ranges, whereas categorical and count data are presented as frequencies and percentages.

OS was defined as the time (in months) from the date of surgery until the last follow-up or date of death. LCSS was defined as the time (in months) from the date of surgery until the last follow-up or date of recurrence. OS and LCSS were calculated using Kaplan–Meier estimates. Statistical analyses were performed using JMP^®^ 14 (SAS Institute Inc., Cary, NC, USA).

## RESULTS

We included eight patients (cases 1–8) who underwent CL after anatomical segmentectomy in the same lobe from January 2005 to December 2019. The baseline characteristics before CL are summarized in Table 1, and the outcomes of CL are summarized in Table 2. Details of the individual cases are shown in Table 3.

**Table 1:** Characteristics of the patients before completion lobectomy

Variable	*N* = 8
Age (years)	69.0
Median (IQR)	(64.2–74.2)
Sex, *n* (%)	4 (50.0)/4 (50.0)
Male/female	
ASA score Median (IQR)	2 (1–2)
Charlson comorbidity index	1
Median (IQR)	(0–3.5)
FVC, L	
Median (IQR)	2.6 (2.1–3.3)
FEV1.0 (l) Median (IQR)	1.8 (1.4–2.5)
Interval between segmentectomy and completion lobectomy (months) Median (IQR)	24 (1.9–30.3)
Lymph node dissection at segmentectomy, *n* (%) ND0/ND1/ND2	5 (62.5)/2 (25.0)/1 (12.5)
Approach in first operation VATS/Thoracotomy, *n* (%)	6 (75.0)/2 (25.0)
Fibrin glue at first operation, *n* (%) +/−	6 (75.0)/2(25.0)
Coverage of the segmental plane, *n* (%) Suture/covering sheet/staple/none	4 (50.0)/2 (25.0)/1 (12.5)/1 (12.5)

ASA: American Society of Anesthesiologists; FEV: forced expiratory volume; FVC: forced vital capacity; IQR: interquartile range; ND: nodal dissection; VATS: video-assisted thoracoscopic surgery.

**Table 2:** Outcomes of completion lobectomy

Procedure in second operation VATS/Thoracotomy, *n* (%)	3 (37.5)/5 (62.5)
Conversion to thoracotomy from VATS	0
Site of lobectomy, *n* (%) RUL/RML/RLL LUL/LLL	1 (12.5)/0 (0)/4 (50.0) 2 (25.0)/1 (12.5)
Bleeding, g	251
Median (IQR)	(212.0–815.2)
Operative time (min)	205
Median (IQR)	(167.5–232)
Lymph node dissection at completion lobectomy, *n* (%) ND0/ND1/ND2	1 (12.5)/0 (0)/7 (87.5)
Postoperative histology, *n* (%) Primary lung cancer/metastasis/granuloma	6 (75.0)/1 (12.5)/1(12.5)
Postoperative hospitalization duration (days)	6
Median (IQR)	(5-7)
Postoperative drainage duration (days)	1
Median (IQR)	(1)
Follow-up time, months	66.9
Median (IQR)	(26.9–83.1)
Clavien–Dindo grade II+ complication, *n*	2
30-day mortality, *n*	0

IQR: interquartile range; LLL: left lower lobe; LUL: left upper lobe; ND: nodal dissection; RLL: right lower lobe; RML: right middle lobe; RUL: right upper lobe; VATS: video-assisted thoracoscopic surgery.

**Table 3:** Details of individual cases after completion lobectomy

No.	Reason for CL	Diagnosis	Interval to CL, weeks	CL	ND	Second operative approach	Operative time, min	Bleeding, g	Severity of adhesions	PA injury	Taping of main PA	Postoperative drainage duration, days	Hospital stays after surgery, days	Clavien–Dindo grade II+ complication	30-day mortality	Follow-up period, months	Reason for death
1	Second LC	LC	306	LUL	2	Thoracotomy	194	543	Severe	–	+	1	10	–	–	86.3	–
2	second MC	MC	98	RUL	2	VATS	234	61	Moderate	–	+	1	5	Arrhythmia G2	–	1.7	Suicide
3	Pathological change	LC	1	RLL	2	VATS	138	253	Moderate	–	–	1	6	–	–	97.9	–
4	Recurrence of staple line	Granuloma	109	RLL	2	Thoracotomy	165	230	Severe	+	–	1	5	–	–	68	Unknown
5	Pathological change	LC	4	RLL	2	Thoracotomy	226	906	Severe	+	+	6	6	Air leakage G3a	–	39.3	Pneumonia
6	Pathological change	LC	19	LLL	2	VATS	216	206	Severe	–	–	1	6	–	–	73.5	–
7	Second LC	LC	120	RLL	2	Thoracotomy	175	248	Moderate	–	+	1	8	–	–	65.9	–
8	Second LC	LC	133	LUL	0	Thoracotomy	407	2194	Severe	–	–	1	7	–	–	22.8	–

LC: lung cancer; LLL: left lower lobe; LUL: left upper lobe; MC: metastatic cancer; ND: nodal dissection; PA: pulmonary artery; RLL: right lower lobe; RUL: right upper lobe; VATS: video-assisted thoracoscopic surgery.

The median postoperative hospitalization duration was 6 days (interquartile range, 5–7 days). Postoperative complications (Clavien–Dindo grade ≥II) occurred in 2 patients, with arrhythmia (Clavien–Dindo grade II) in one and air leakage (Clavien–Dindo Grade IIIa) in the other. No postoperative mortality or recurrence of lung cancer was observed. All the patients with lung cancer were alive and recurrence-free at the time of publication. The 5-year LCSS rate was 100% (95% confidence interval 100–100), and the 5-year OS was 72.9% (95% confidence interval 34.3–93.2).

The indications for the first operation comprised a clinical diagnosis of lung cancer or metastatic lung tumour in 6 patients, and inflammatory cysts and mucosa-associated lymphoid tissue lymphoma in one patient each. None of the patients had a preoperative pathological diagnosis. The indications for the second operation were second primary lung cancer in 3 patients, second metastasis and suspected recurrence of the tumour in the staple lines in one patient each and a change in the pathological diagnosis from the intraoperative frozen-section diagnosis in 3 patients (initial diagnosis: benign, metastatic and low-grade malignant tumour in one patient each; final diagnosis: primary lung cancer in all 3 patients). The final pathological diagnosis was primary lung cancer in 6 patients, secondary metastasis in 1 patient and granuloma in 1 patient. The patient with suspected recurrence in the staple lines was finally diagnosed with a granuloma.

### Cases of pulmonary artery injury

#### Case 4

The patient underwent right S10 segmentectomy through thoracotomy via a posterior approach. The PA was approached from the interlobar fissure, and we attempted to tunnel the interlobar fissure between the right middle and lower lobes. However, a small PA injury occurred due to severe hilar adhesion, but it was controlled well using compression haemostasis. After the haemostasis, we divided the pulmonary vein (PV) and bronchus. We simultaneously divided the PA and lung parenchyma using a stapler. The amount of bleeding in this case was 230 g.

#### Case 5

The patient underwent right S6 segmentectomy, and the pathological diagnosis differed from the intraoperative frozen-section diagnosis. In the initial surgery, we injured (and repaired) the bronchus intermedius due to the severely inflamed lymph nodes around the bronchus intermedius. In the CL, the PA was approached from the interlobar fissure, and we were able to divide A7–8; however, the interlobar fissure surrounding A9–10 was scarred and could not be identified, and a PA injury occurred due to severe hilar adhesions and scarring. We taped the main PA. The area around the scar and the PA (A9–10) was clamped. We incised it with scissors and sutured the stump step by step. Finally, we divided the bronchus using a stapler. The amount of bleeding was 906 g. No blood transfusion was required.

### Cases of left upper lobe lobectomy

#### Case 1

The patient underwent left S1 + 2 segmentectomy with mediastinal lymph node sampling (#5 and #10 dissection). In the initial surgery, the segmental plane was covered with a polyglycolic acid (PGA) sheet. There were severe adhesions at the hilum (especially around the A1 + 2 stump) and chest wall due to the PGA sheet and mediastinal lymph node dissection. We taped the main PA and superior PV in the pericardium. The management of the PA around A3 differed, as A4 + 5 dissection was possible. We divided the superior PV in the pericardium. Subsequently, we divided the bronchus. The scars and severe adhesions were carefully dissected around the PA. Finally, we simultaneously divided the PA (A3, 1 + 2) and lung parenchyma using a stapler.

#### Case 8

The patient underwent left S1 + 2 + 3 segmentectomy combined with wedge bronchoplasty and S6 + 9a + 10a segmentectomy (nodal dissection 2a-2) for synchronous lung cancers via thoracotomy. Two years later, a new lesion was found in his lingular segment, and a second operation was performed. In the second operation via thoracotomy, there were tight adhesions in the thoracic cavity, and we performed extrapleural dissection. We attempted to tape the PA and PV in the pericardium but were unsuccessful because of inflammatory adhesions in the pericardium due to the initial surgery. We were able to perform left upper lobectomy with careful manipulation of the hilum. The PA was not injured; however, there were multiple lung injuries and chest wall injuries, resulting in a substantial amount of bleeding. However, no blood transfusion was required.

### Cases using video-assisted thoracoscopic surgery as the second operative procedure

#### Case 2

The patient underwent right 3a subsegmentectomy. In the initial surgery, we approached from the interlobar fissure and dissected A3a and B3a. The segmental plane was closed by running sutures using monofilament 4-0 proline thread. In the secondary surgery, severe adhesions were observed around the truncus pulmonalis. We taped the main PA in the pericardium. The scars and adhesions surrounding the truncus pulmonalis were thoroughly dissected using a stapler.

#### Case 3

The patient underwent right 8a segmentectomy. In the initial surgery, we used an interlobar fissure approach and dissected A8a and B8a. The segmental plane was sutured to prevent adhesions. In the secondary surgery, although there were hilar adhesions, we were able to perform adhesiolysis on the central side of A8 and dissected A6–10.

#### Case 6

The patient underwent left 9 + 10 segmentectomy. In the initial surgery, we used a posterior approach, but the PA was divided from the interlobar fissure. The second surgery was delayed due to a delay in receiving the pathology test results. Tight adhesions and cicatrization were observed around the PA; however, the space between the bronchus and PA was free from such adhesions. After tunnelling the space between the bronchus and PA, we simultaneously divided the PA (A6, 8) and lung parenchyma using a stapler. The severe adhesions around the descending aorta were divided using a stapler.

### Cases using open thoracotomy as the second operative procedure (and video-assisted thoracoscopic surgery as the first operative procedure)

#### Case 1

As mentioned above, the patient underwent left S1 + 2 segmentectomy, with mediastinal lymph node sampling (#5 and #10 dissection). In the initial surgery, we covered the segmental plane with a PGA sheet. We predicted difficulties due to adhesions; therefore, we performed thoracotomy.

#### Case 5

As mentioned above, in the initial surgery, we injured (and repaired) the bronchus intermedius due to the severely inflamed lymph nodes around the bronchus intermedius. In addition, the apex of the lung had severe adhesions due to the effects of post-radiation therapy for the primary disease and was difficult to approach. As we considered dissection around the hilum unfeasible for VATS, we selected thoracotomy.

#### Case 7

The patient underwent right 8 + 6b segmentectomy via VATS. The reason for the second operative procedure by thoracotomy was bronchial stump reinforcement with a pedicled intercostal muscle flap after lower lobectomy.

## DISCUSSION

The present study elucidates the perioperative outcomes of CL after anatomical segmentectomy. Although CL after segmentectomy may show acceptable outcomes, a high degree of adhesion around the hilar and other parts of the lung renders the operation challenging. However, in our cohort, there were no cases of mortality or local recurrence, and the prognosis was good.

Previously, we reported on the use and effectiveness of three-dimensional CT images for surgical simulation during anatomic thoracoscopic pulmonary segmentectomy to secure a sufficient surgical margin. We performed thoracoscopic anatomic lung segmentectomy using three-dimensional CT simulation without tumour markings for non-palpable and non-visualized small lung nodules, and no local recurrence was noted [18–21].

The detection of new solitary pulmonary nodules in a previously resected lobe during postsurgical follow-up, especially after a sublobar resection, poses a diagnostic challenge. CL may be required when the tumour is suspected to be a second primary lung cancer or new metastatic tumour. Moreover, a reoperation, such as CL, may be necessary when the final histopathological diagnosis reveals malignancy in suspected local recurrence cases. As in the present case, there are reports of patients who undergo reoperation for suspected recurrence of the staple line after segmentectomy; however, herein, the tumour was a granuloma [22]. Most sublobar resection cases have been reported to have a thickened staple line. However, staple line thickening on CT may not necessarily indicate tumour recurrence, making it difficult to establish a definitive diagnosis [23]. We believe that pathological confirmation of any suspected recurrence should be obtained with biopsy or surgery. In such cases, diagnosis by biopsy or surgery is necessary. CL is one of the treatments available for patients with local recurrence, a second primary lung cancer and/or metastasis after lung cancer segmentectomy.

CL after segmentectomy is difficult because dissection, exposure and mobilization of the hilum structure are challenging, as the bronchovascular components have already been denuded and manipulated during the previous segmentectomy. Studies have reported that CL may become more difficult if a long period has elapsed since the previous segmentectomy [16, 17]. In addition, CL after superior mediastinal lymph node dissection is especially difficult due to the presence of very severe adhesions [17]. In the present cohort, there was a case of complex segmentectomy (S1 + 2 + 3, S6 + 9a + 10a and bronchoplasty) with superior mediastinal lymph node dissection that resulted in severe adhesions in the thoracic cavity, a long operative time and massive blood loss. CL after anatomical segmentectomy may not be performed safely without fatal complications in patients who have undergone complex surgery. Previous reports have shown that lymph node dissection in the superior mediastinum makes CL more difficult, and Takahashi *et al.* [17] reported massive bleeding in left upper lobectomy after such lymph node dissection. Surgeons may have to be especially careful with complete left upper lobectomy. However, if the PA is difficult to divide due to hilar adhesion, simultaneous PA and lung parenchyma resection may be effective in both thoracotomy and VATS. In some cases, simultaneous dissection of the PA and bronchus may also be considered.

Complications of more than grade II in the Clavien–Dindo classification that were observed in the present study included arrhythmia and air leakage; however, there was no delay in the postoperative discharge and no cases of mortality. Postoperative bleeding and air leakage were minimal, and in all but one patient, the drain could be removed the day after surgery. Although the number of patients is small and several complications occurred in our study, the safety of CL may be considered to be acceptable.

CL after segmentectomy in the same lobe treated using VATS has been reported to be extremely difficult due to the presence of severe hilar adhesions. However, thoracotomy was not required in any cases of CL for planned VATS. In post-segmentectomy surgery, adhesiolysis of the hilum should be performed with an accurate understanding of the anatomy. If the extent or location of the previous operation and adhesions can be predicted, CL can be performed with VATS. However, the previous upper or hilar nodal dissection for upper lesions, mediastinal origin of the lingular artery of the left upper lobe and small distance between the ascending A2 and A6 (or A1 + 2c and A6) may make it difficult to control proximal PA bleeding. Therefore, even if the operator performs the procedure using VATS, one should keep in mind that open thoracotomy may be required, and taping of the PA is important in completion upper lobectomy.

We used fibrin glue or sutures of the visceral pleura in some patients to close the segmental plane (staple line) and PA to prevent adhesions. Adhesions and fibrosis on the staple lines of the intersegmental plane can make the surgery challenging. Moreover, the use of covering material, such as PGA, should be avoided in the segmental plane because it may lead to severe adhesions due to inflammation, rendering CL more difficult [24, 25]. We observed that there were severe adhesions when we used PGA sheets in case 1; thus, these sheets should be used cautiously.

The present study has several limitations. First, the study included a small number of patients. As the study population comprised patients who underwent CL at a single centre, the generalizability of our conclusions to all patients who undergo CL after anatomical segmentectomy requires further investigation. Further, we selected cases for segmentectomy; thus, we were less likely to observe local recurrence. It was difficult to perform CL more frequently. Second, it used a retrospective design. Although it may be difficult to conduct a large-scale and prospective study of CL, many such reports are necessary to acquire robust evidence to support the safety of the procedure. Although each intrathoracic or hilar adhesion rendered the surgery difficult, it was possible to perform CL, supporting its feasibility.

## CONCLUSIONS

CL after anatomical segmentectomy for lung cancer in the same lobe can be performed without fatal complications in selected patients. When the PA is difficult to divide due to hilar adhesions, simultaneous PA and lung parenchyma resection is effective in both thoracotomy and VATS.

## ABBREVIATIONS

CL: Completion lobectomy
CT: Computed tomography
LCSS: Lung cancer-specific survival
NSCLC: Non-small-cell lung cancer
OS: Overall survival
PA: Pulmonary artery
PGA: Polyglycolic acid
PV: Pulmonary vein
VATS: Video-assisted thoracoscopic surgery
